# Posterior parasagittal meningiomas display aggressive features independent of size: a multicenter analysis

**DOI:** 10.1007/s11060-025-05103-z

**Published:** 2025-06-26

**Authors:** Said Koçyiğit, Miguel Millares Chavez, Ömer Orhun, Joseph O’Brien, Arda Inan, A. Harun Yaşar, Alp Dinçer, Jennifer Moliterno, Murat Günel, M. Necmettin Pamir, Koray Özduman, Ayça Erşen-Danyeli

**Affiliations:** 1 School of Medicine, Department of Neurosurgery, Acıbadem University, Istanbul, Türkiye; 2 School of Medicine, Department of Neurosurgery, Yale University, New Haven, USA; 3 School of Medicine, Department of Pathology, Acıbadem University, Altunizade, Yurtcan Sokağı No:1, 34662 Üsküdar, Istanbul, Türkiye; 4 School of Medicine, Department of Radiology, Acıbadem University, Istanbul, Türkiye

**Keywords:** Meningioma, Tumor size, Atypical, Grade, Location, Radiologic predictors

## Abstract

**Purpose:**

While meningioma size is known to correlate with higher histological grade, tumor behavior can vary by anatomical location, suggesting that some meningiomas may exhibit aggressive features early and independent of size. We hypothesized that posterior parasagittal meningiomas possess unique growth characteristics and tested this hypothesis using a retrospective analysis of two independent cohorts.

**Methods:**

Cohort-A (*n* = 316) included 123 WHO-grade 2 (GR2) and 193 age and location matched WHO-grade 1 (GR1) meningiomas. Twelve radiological features as well as the histological subtypes, histological grading features and 1p status was evaluated. A volume index (V_i_), defined as the GR2/GR1 tumor volume ratio, was calculated across different anatomical locations. Findings were validated in Cohort-B (*n* = 477), which also included NF2-driven and non-NF2-driven molecular subsets.

**Results:**

Tumor volume correlated strongly with GR2 status (*p* = 3.5 × 10^− 6^) and histopathological markers of major grading criteria including mitotic count (*p* < 0.001), brain invasion (*p* < 0.05), and minor grading criteria including hypercellularity (*p* < 0.001), necrosis (*p* < 0.001) along with increased Ki67 index (*p* < 0.01). Anatomically, the non-skull base posterior midline (NSB-POST-M) meningiomas had the lowest V_i_ in both cohorts and the NF2-driven subset indicating that these tumors exhibit aggressive features even at smaller sizes. The NSB-POST-M location had the highest proportion of GR2 cases, mean Ki67 index, and incidence of chromosome 1p loss.

**Conclusions:**

While larger meningiomas are generally more aggressive, posterior parasagittal meningiomas display aggressive biology regardless of size. These findings suggest that anatomical location should be incorporated into risk stratification and management decisions.

## Introduction

Meningiomas are the most common primary brain tumors [1]. Their recurrence risk and treatment strategies are primarily guided by the histopathological grade, which remains the most widely used predictor of aggressive behavior [2]. Larger meningiomas are typically associated with higher WHO grade, likely reflecting the accumulation of genetic alterations over time [3, 4]. However, meningiomas are a heterogenous group of tumors whose behavior is influenced not only by size but also by anatomical location and molecular profile [4].

Previous studies have reported regional differences in tumor size at diagnosis, with some locations favoring smaller tumors due to neuroanatomical constraints [5]. Moreover, emerging molecular classification of meningiomas demonstrate that different genetic drivers can lead to distinct tumor behavior based on anatomical site [4, 6–9]. These findings raise an important question: Is the correlation between tumor size and grade consistent across all brain regions, or does it vary by anatomical location?

We hypothesized that non-skull base, posterior parasagittal and falx meningiomas exhibit aggressive biological behavior independent of tumor size. To test this hypothesis, we conducted a three-step analysis using two independent cohorts.

## Materials and methods

### Study design

**Fig. 1:**
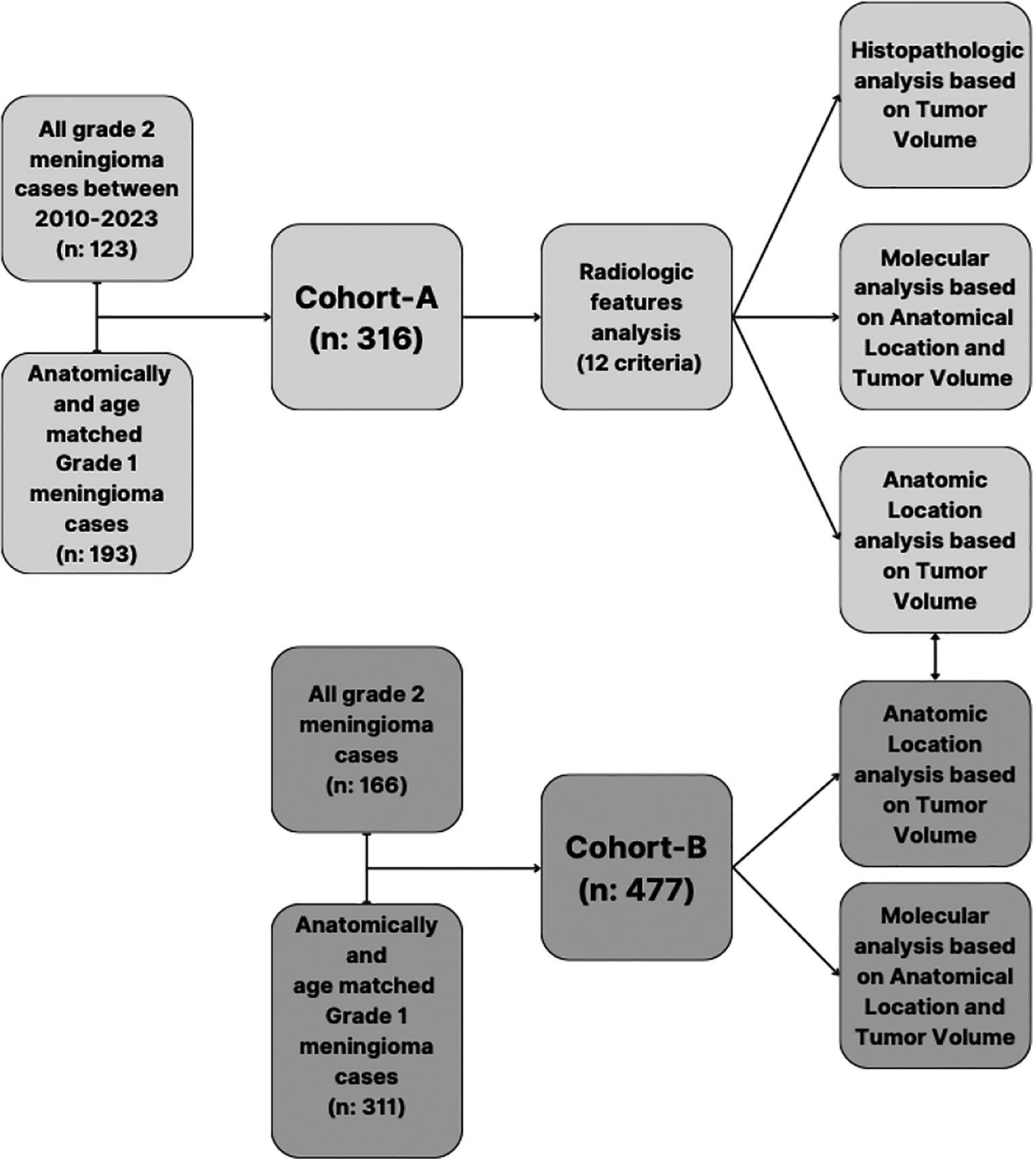
Study protocol. This figure outlines the study design, representing the data collection and inclusion criteria, step by step analysis of correlation between anatomical location and tumor volume together with radiological and histopathological characteristics of meningiomas, in two cohorts

We performed a retrospective analysis using two independent cohorts (Cohort-A and Cohort-B) to evaluate the relationship between tumor size, histological grade, anatomical location and molecular characteristics. The analysis consisted of three analytical steps (Fig. 1):

1. Validation of the established correlation between tumor size and GR2 status.
2. Assessment of histopathological and molecular correlates of aggressive tumor behavior.
3. Evaluation of anatomical variations in the volume index (Vi) and their consistency across cohorts.

### Patient selection

Cohort-A (*n* = 316) consisted of 123 (39%) GR2 and 193 (61%) age and location matched GR1 meningiomas. All GR2 meningiomas operated between 2010 and 2023 were retrospectively identified from medical archives. Patients with a history of meningioma surgery, meningioma radiosurgery, or meningioma radiotherapy and those with another neurological disease were excluded. 193 age and anatomical location-matched GR1 patients were selected from the same time interval.

Cohort-B (*n* = 477) served as an external validation set. Similar exclusion criteria were applied to this cohort. Ethics approval was granted by Acıbadem University Ethics Committee (ATADEK, approval number 2024-18/7). 

**Fig. 2:**
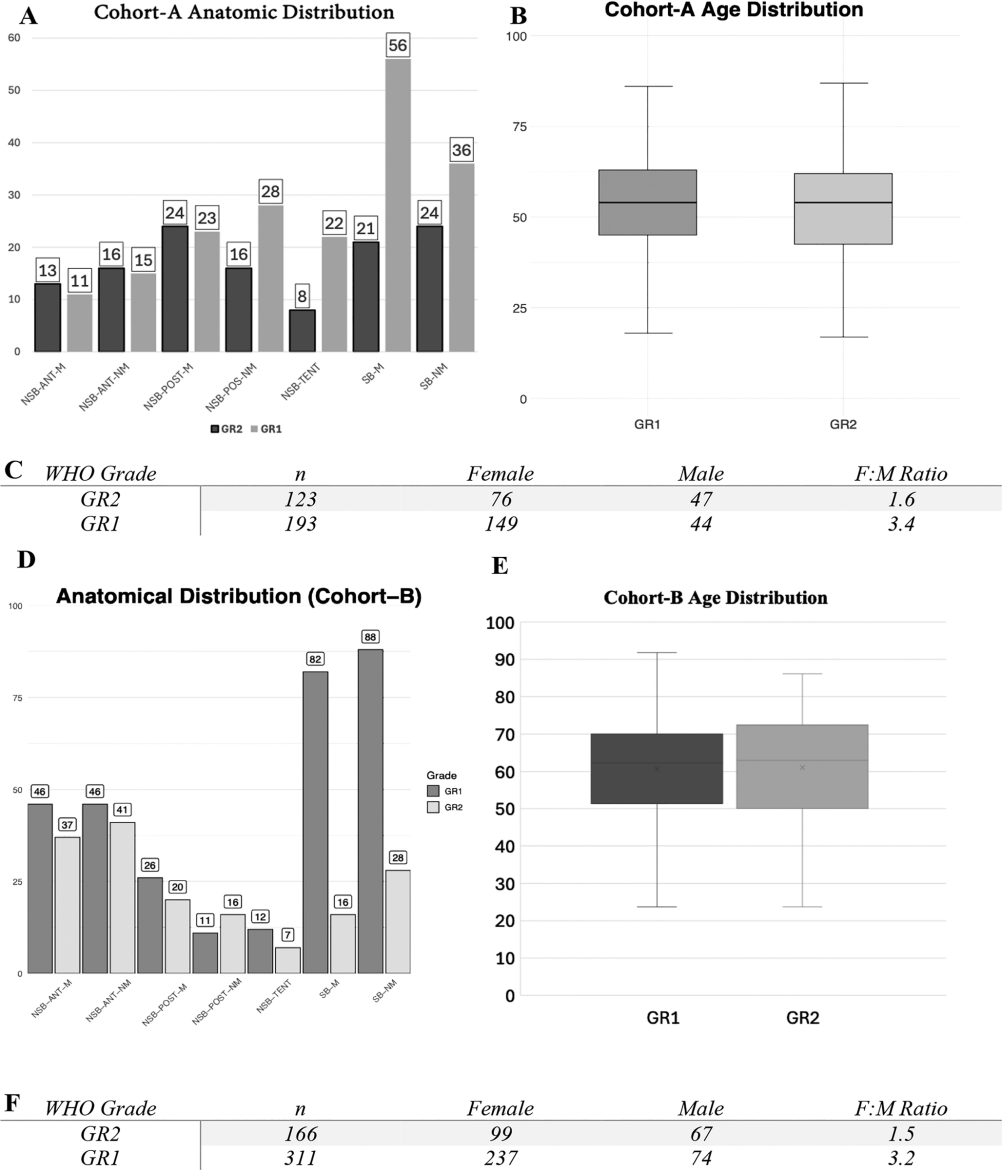
The demographic and anatomical distribution of meningiomas within 2 cohorts. Panels show the anatomical locations (**A**), age distribution (**B**), and gender distribution (**C**) of Cohort-A. Second part of the figure shows anatomical locations (**D**), age distribution (**E**), and gender distribution (**F**) of Cohort-B

Details of each cohort is presented in Fig. 2: In Cohort-A GR2 cases had a mean age of 53 (SD: +/-14.3) and GR1 cases 54 (SD: +/-12.6) (difference statistically insignificant; *p* = 0.4782). Female: male ratio was 1.6 in GR2 and 3.4 in GR1. 46% (*n* = 137) of meningiomas were located in the skull base, 43% (*n* = 146) in the falx/parasagittal or convexity, and 10% (*n* = 30) in the tentorial region. Cohort-B consisted of 477 meningioma patients. 311 (65%) were GR1 and 166 (35%) were GR2. The mean age was 61 (SD:+/-13.5) for Cohort-B, 61 (SD:+/-14.5) for GR2 and 61 (SD:+/-12.9) for GR1 patients. The female-to-male ratio was 2.4 for Cohort-B, 1.5 for GR2, and 3.2 for GR1 patients.

### Radiological analysis

Tumor volume and 12 semantic radiological features were assessed by two observers (S.K. and A.D.) through consensus. These features were (1) irregular tumor margins [lobulation], (2) presence of [necrosis/hemorrhage], (3) [peritumoral edema], (4) [intratumoral heterogeneity] in hyperintense MRI signal, (5) [mass effect], (6) [location] skull base/convexity, (7) [cystic component], (8) [intratumoral calcification], (9) [dural tail], (10) [hyperostosis at base], 11) [en-plaque growth pattern], and 12) the [tumor volume] were correlated with GR2 diagnosis. Pearson’s chi-square test and logistic regression were used for statistical analysis.

### Pathology and molecular genetic analyses

Pathology slides from Cohort-A were retrospectively re-evaluated according to the WHO 2021 criteria including major (brain invasion and mitotic count) and minor (hypercellularity, necrosis, sheeting, macronucleoli, small cell change) grade 2 criteria along with Ki67 proliferation index by two neuropathologists (A.E.D. and A.I.) [10]. The Ki67 proliferation index (using the MIB-1 monoclonal antibody, Agilent Cat# M724001-2, RRID: AB_2631211) was scored by digital image analysis (ViraPath software, Virasoft, Istanbul, Turkey) [6]. Chromosome 1p deletion status was assessed in Cohort-A using the Infinium Methylation EPIC v2.0 array (Illumina, San Diego, CA) and interpreted using (Heidelberg/DKFZ online Brain Tumor Classifier version 12.8) [11].

### Anatomical locations

Tumor locations were obtained from operative and radiology reports and categorized based on the classification system proposed by Youngblood et al. [4]. For analytical purposes, we grouped skull base tumors into two simplified categories: SB-M (Skull Base–Midline), comprising SB-AF-M (anterior fossa midline), SB-MF-M (middle fossa midline), and SB-PF-M (posterior fossa midline); and SB-NM (Skull Base–Non-Midline), comprising SB-AF-NM, SB-MF-NM, and SB-PF-NM. The remaining tumors were classified using the original categories defined by Youngblood et al.: NSB-ANT-M (Non-Skull Base Anterior Midline), NSB-ANT-NM (Non-Skull Base Anterior Non-Midline), NSB-POST-M (Non-Skull Base Posterior Midline), NSB-POST-NM (Non-Skull Base Posterior Non-Midline), NSB-TENT (Non-Skull Base Tentorial). Cases outside these anatomical locations were not included in the volume index comparison. This approach was used to balance anatomical detail with statistical power in subgroup analyses (see Fig. 2a).

One outlier in Cohort B was excluded due to an extremely high tumor volume (107,17cm3). The volume index (Vi), defined as the GR2/GR1 tumor volume ratio was used to assess regional growth behavior.

### Statistical analysis

Statistical comparisons were conducted using chi-square tests for categorical variables and Welch’s t-tests for continuous variables. The volume index (Vi) was calculated to normalize for anatomical constraints and statistical comparisons conducted utilizing ANOVA. Statistical significance was defined as *p* < 0.05. All analyses were performed in R Studio (Version 2023.12.1 + 402).

## Results

### Size-grade correlation and histopathological findings

**Fig. 3:**
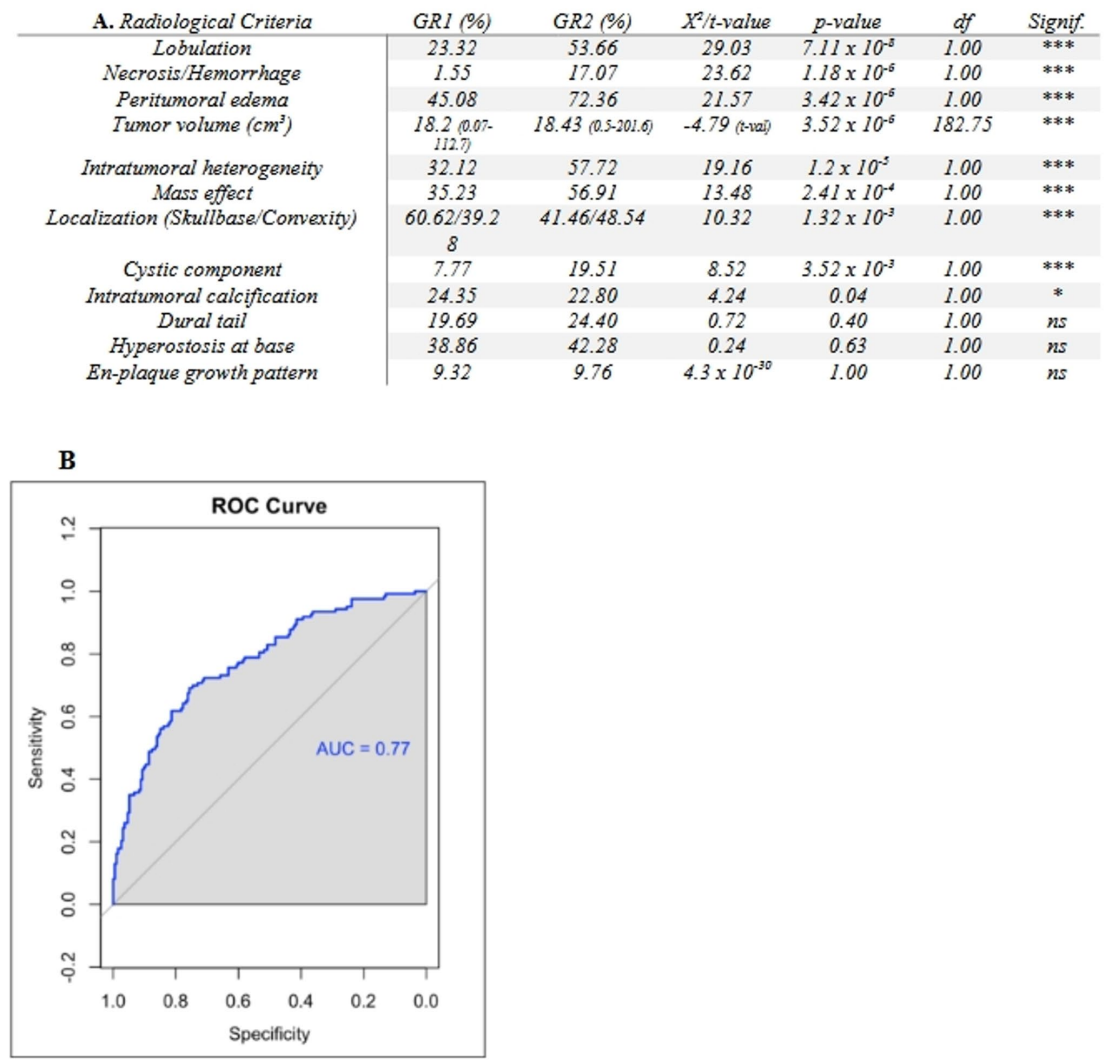
The difference in MRI-derived radiological features between GR2 and GR1 meningiomas. A chi-square analysis displaying the correlation between certain semantic MRI features and the likelihood of GR2 classification, sorted based on their significance level (**A**). An area under the curve (AUC) analysis from univariate logistic regression, indicating the predictive value of radiological characteristics in identifying high-grade tumors (**B**)

In Cohort-A, mean tumor volume was significantly higher in GR2 than in GR1 meningiomas (40.84+/-31.98 cm^3^ vs. 22.18+/-30.1cm^3^, *p* = 2.432 10^− 7^). Among the 12 radiological features assessed, 9 (75%) showed significant association GR2 diagnosis (Fig. 3a). The AUC for the univariate logistic regression predicting GR2 was 77% and 9/12 (75%) of the correlations reached significance.

**Fig. 4:**
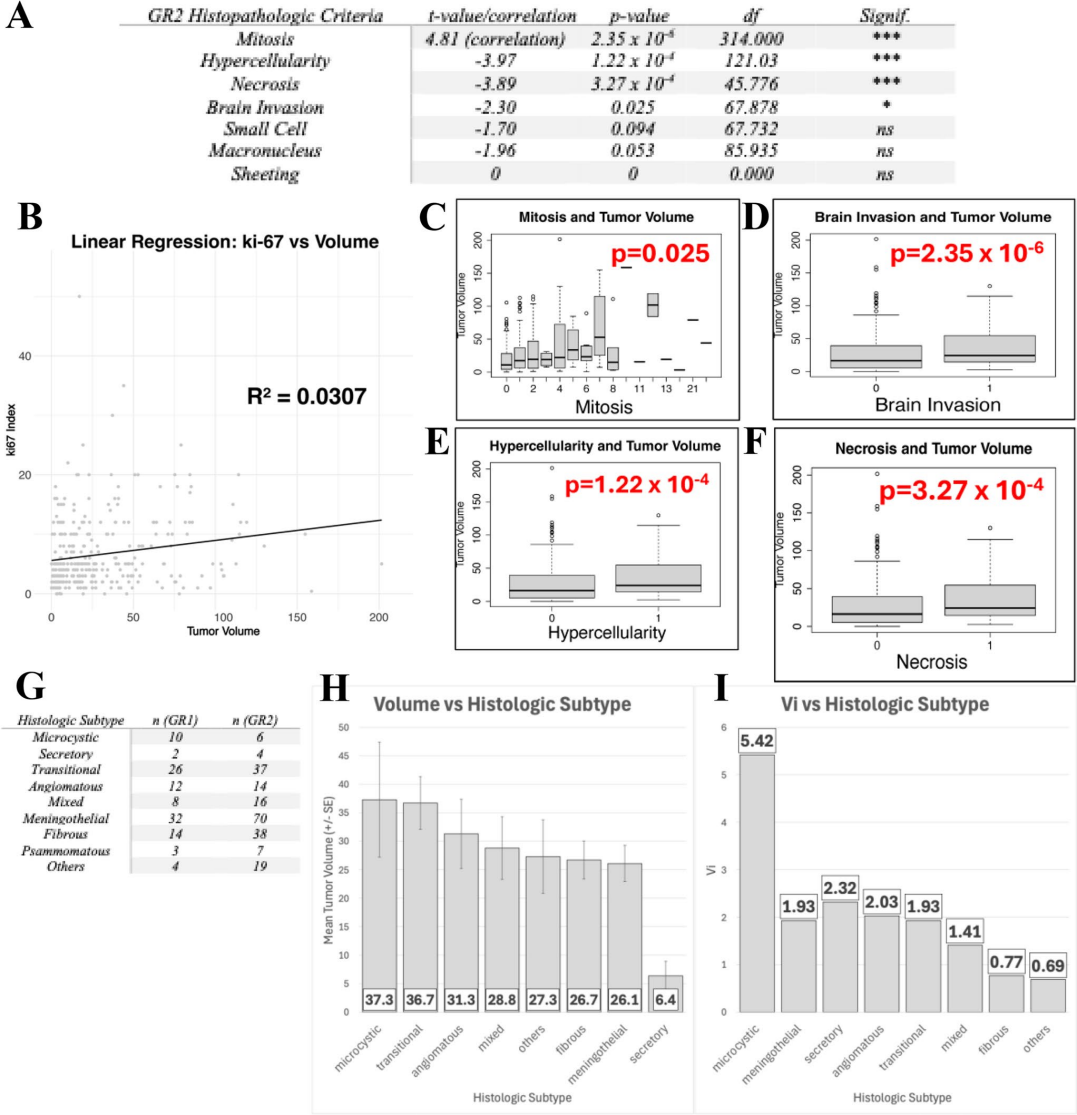
The correlation of tumor volume with histopathological criteria and histologic subtypes. Histopathological features, associated with WHO grading, such as high mitotic index, increased Ki67 proliferative index, hypercellularity, and necrosis, demonstrate a positive correlation with tumor volume, suggesting that larger meningiomas are more likely to exhibit aggressive biological behaviour (**A**-**F**). The number of cases for each subtype presented in (**G**). The data reveal that microcystic and meningothelial subtypes, known to have benign tumor biology, have significantly larger tumor volumes compared to other variants (**H**), (**I**). These findings align with the hypothesis that benign tumors require a greater number of cell divisions, and thus, more genetic hits, to achieve substantial growth

**Fig. 5:**
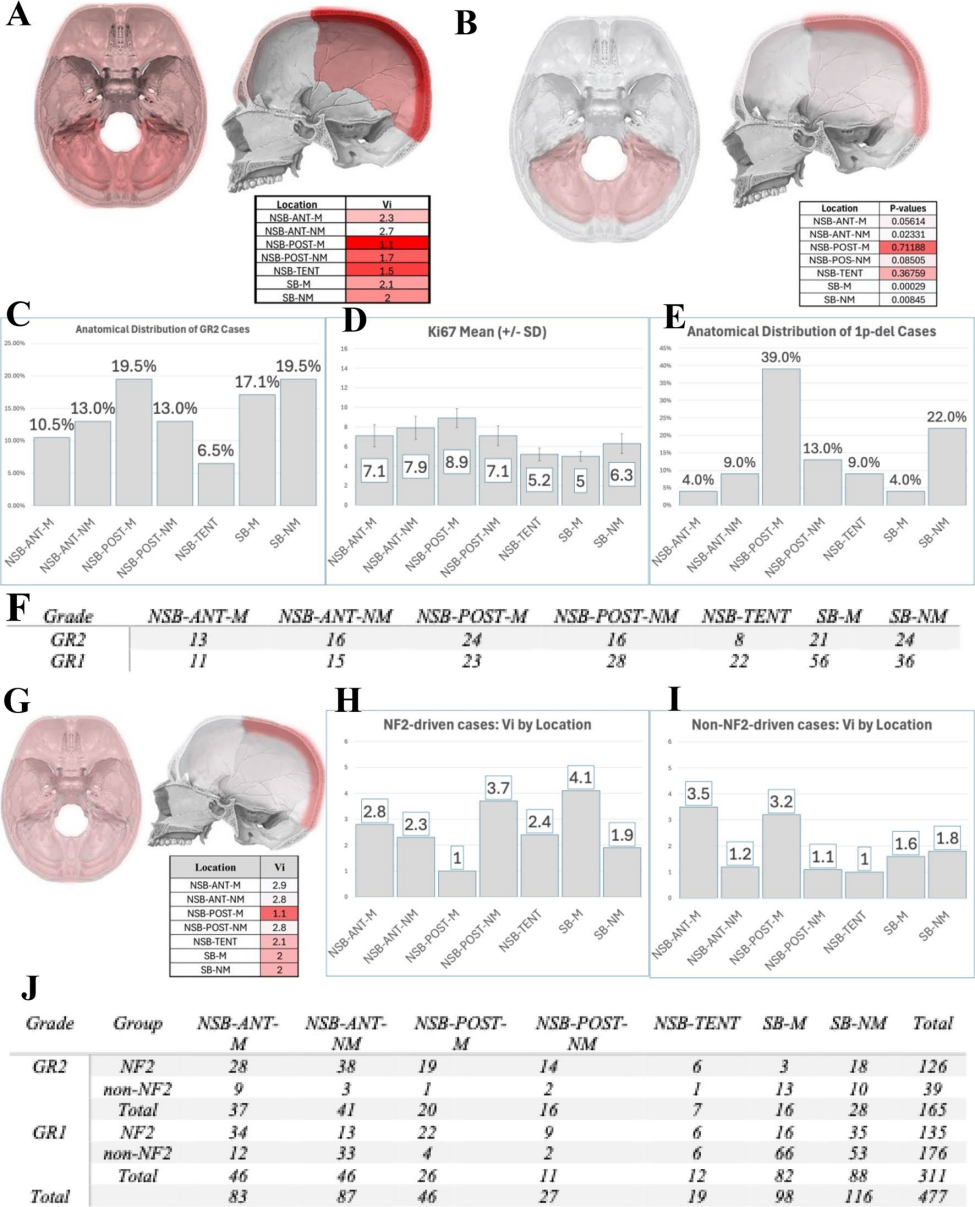
Posterior parasagittal meningiomas display aggressive features and display no significant difference between GR1 and GR2. The volume index (VI) is lowest for the NSB-POST-M localization (**A**), indicating that there is little volume difference between GR1 and GR2 meningiomas. The volumetric difference between GR1 and GR2 cases is not significant in this localization where the “p value” is the highest among all locations (**B**, t-test) Findings that point to an aggressive tumor biology are: The highest percentage of GR2 cases (**C**), highest Ki-67 proliferative index (**D**), highest percentage of Chromosome 1p loss (**E**) for NSB-POST-M meningiomas. Table in (**F**) indicates the number of cases of GR1 and GR2 cases at all anatomical locations. The replication of the V_i_ findings on the Cohort-A to an independent Cohort-B, ensuring reproducibility and validation of findings. The volume index (V_i_) is the lowest for the NSB-POST-M localization (**G**). The presence of an NF2 mutation is associated with the lowest volume index in NSB-POST-M localization, suggesting a distinct growth pattern between NF2-driven and non-NF2-driven meningiomas in this region (**H**), (**I**). Table summarizes the number of GR1 and GR2 cases for each anatomical location (J)

Tumor size was positively associated with histopathological features including mitotic index (*p* < 0.001), hypercellularity (*p* < 0.001), necrosis (*p* < 0.001) and brain invasion (*p* = 0.025). The Ki67 mitotic index also correlated strongly with tumor volume (R^2^ = 0.0307, *p* = 1.78 × 10^− 3^) (Fig. 5d). Correlations of all major and minor histopathological criteria for GR2 diagnosis are present in Fig. 4.

When histological subtypes were stratified by Vi, the lowest values were found in the “others” subgroup (chordoid, metaplastic, clear cell, and atypical subtypes) due to their low case counts, followed by the fibrous histology. Vi values for all histological subtypes are presented in Fig. 5.

### Regional variation in the size-grade relationship

Anatomical analysis revealed that the NSB-POST-M location had the lowest Vi across both cohorts (both 1.1 in Cohort-A and in Cohort-B), indicating that GR2 tumors in this region present at similar sizes to GR1 tumors. Despite their smaller size, NSB-POST-M meningiomas had the highest GR2 proportion (19.5%), mean Ki67 index (8.9%), and chromosome 1p loss rate (39%) (Fig. 5).

### Molecular correlations of the regional variation

Among NF2-driven meningiomas, NSB-POST-M tumors maintained the lowest Vi (1), suggesting that anatomical influence on aggressive growth patterns is preserved in this molecular subtype. In contrast, non-NF2-driven tumors did not exhibit this pattern (Fig. 5).

## Discussion

Our findings confirm that larger meningiomas are generally associated with higher WHO grade [3, 11, 12]. Tumor volume correlated with histopathological markers of aggressive behavior, including Ki-67 index, mitotic activity, hypercellularity, necrosis, and brain invasion. These results support the hypothesis that meningiomas become more aggressive with successive cell divisions.

However, we also found that posterior parasagittal meningiomas exhibit more aggressive features even at smaller sizes. Specifically, NSB-POST-M tumors, which corresponds to the parasagittal/parafalcine meningiomas posterior to the coronal suture, exhibited more aggressive features irrespective of the tumor volume. This pattern, consistent across both independent cohorts, challenges the long-standing assumption that tumor size alone reliably predicts meningioma grade. Several biological mechanisms may underlie this phenomenon:

First, there is clear evidence of regional differences in driver mutations. Anterior parasagittal meningiomas are often driven by *TRAF7*, *KLF4*, *or SMO* mutations, while posterior ones are typically NF2-driven [8]. NF2-driven meningiomas have been associated with a more aggressive histology and earlier recurrence [4, 6–9].

Second, genetic instability inherent to posterior parasagittal meningiomas may result in tumors that are aggressive while still small. Prior comprehensive molecular genetic analyses have identified parasagittal meningiomas as genetically unstable [14–17]. Posterior parasagittal meningiomas had the highest incidence of chromosome 1p loss, which is currently considered the earliest finding in meningioma genetic instability and criteria for higher grade tumors [18] This molecular vulnerability could predispose even small tumors in this location to exhibit atypical or anaplastic features, without the need for a high number of cell divisions.

Third, the embryologic origin of the meninges may also influence tumor behavior. Posterior parasagittal meninges are believed to arise from mesenchymal progenitors, whereas anterior regions may be derived from neural crest cells [19]. This distinction may underlie spatial variations in tumor biology. Our previous finding of a ventro-caudal decreasing gradient of epithelial vs. mesenchymal markers reinforce the idea that parasagittal/falx meningiomas follow a spatial gradient rather than a categorical division [4, 6].

Lastly, we acknowledge that anatomical constraints can limit tumor expansion and influence the timing of clinical detection. To account for this, we used the volume index (Vi) to normalize for anatomical constraints on tumor size, allowing a more accurate comparison of tumor behavior across regions.

These findings carry clinical implications. Current surveillance protocols, which often consider size a primary risk factor, may underestimate the risk associated with posterior midline meningiomas [20, 21]. Recognizing high-risk behavior in a small meningioma has potential to influence decisions on surveillance, surgical timing, aggressivity of treatment and follow-up and can also guide patient counselling, especially when discussing treatment options for small but high-risk tumors. Surveillance strategies may need to be adjusted and even small incidental meningiomas in this location may warrant shorter imaging intervals and closer monitoring for subtle growth or early radiologic signs of aggressiveness (e.g., edema, invasion, heterogeneity). Similarly surgical timing may need reconsideration based on the location of the meningioma: The traditional “ watchful waiting” approach commonly applied to small, asymptomatic tumors may not be appropriate for posterior midline lesions. Early surgical or radiosurgical intervention could potentially prevent progression to a more aggressive phenotype. In addition, early resection might be pursued not only for symptomatic or radiographic growth, but to obtain early histopathological and molecular diagnosis. Finally, these findings may also influence treatment decisions. Knowledge of the location-specific risk can guide neurosurgeons and oncologists in discussions of extent of resection, the need for adjuvant radiotherapy, and more aggressive treatment plans—even for relatively small lesions. Taken together, our findings suggest that anatomical location—particularly NSB-POST-M positioning—should be incorporated into future clinical guidelines alongside tumor size and WHO grade.

While our study provides evidence that posterior parasagittal meningiomas exhibit aggressive behavior, a survival analysis was not in the scope of this analysis. Similarly, a comprehensive molecular analysis for a mechanistic explanation, why the posterior parasagittal region has more aggressive behavior is under study. Future studies should investigate whether early intervention in these tumors improves patient outcomes.

## Conclusion

Tumor size can be associated with WHO grade in meningiomas; however, this relationship appears to vary with anatomical location. Our findings demonstrate that posterior parasagittal meningiomas can exhibit more aggressive biological features– including higher mitotic index, increased Chr1p loss, and greater GR2 prevalence- even at smaller sizes. These results highlight the need to consider anatomical location as an independent factor in meningioma risk stratification and treatment planning. Incorporating location into clinical decision-making may improve early detection of high-risk tumors, guide surgical timing, and inform the use of adjuvant therapies, particularly for smaller tumors located in the posterior midline.

## Data Availability

No datasets were generated or analysed during the current study.

## Abbreviations

WHO: World Health Organization
GR1: World Health Organization Grade 1 Meningioma
GR2: World Health Organization Grade 2 Meningioma
MRI: Magnetic Resonance Imaging
V_i_: Volume Index
NSB-ANT-M: Non-Skull Base Anterior Midline
NSB-ANT-NM: Non-Skull Base Anterior Non-Midline
NSB-POST-M: Non-Skull Base Posterior Midline
NSB-POST-NM: Non-Skull Base Posterior Non-Midline
NSB-TENT: Non-Skull Base Tentorial
SB-M: Skull Base Midline
SB-NM: Skull Base Non-Midline
R²: Coefficient of Determination
AUC: Area Under the Curve
ANOVA: Analysis of Variance
Chr1p: Chromosome 1p
NF2: Neurofibromin 2 gene
TRAF7: Tumor Necrosis Factor Receptor Associated Factor 7
KLF4: Kruppel-Like Factor 4
SMO: Smoothened gene
DKFZ: Deutsches Krebsforschungszentrum (German Cancer Research Center)
